# Patch-based, iteratively-reweighted compressive recovery for reconstruction of highly accelerated exercise stress cardiac cine

**DOI:** 10.1186/1532-429X-18-S1-P331

**Published:** 2016-01-27

**Authors:** Samuel T Ting, Rizwan Ahmad, Ning Jin, Orlando P Simonetti

**Affiliations:** 1 grid.261331.40000000122857943The Ohio State University, Columbus, OH USA; 2 Siemens Healthcare, Columbus, OH USA

## Background

Real-time exercise stress cardiac magnetic resonance imaging is challenging due to exaggerated breathing motion and high heart rates; improvements in image reconstruction may help improve the reliability and diagnostic accuracy of this difficult imaging application. Cardiac images possess a rich structure that can be exploited to aid image reconstruction by enforcing sparsity in an appropriate transformed domain, e.g., in the undecimated wavelet transform (UWT) domain). When using UWT or its decimated counterpart, standard techniques achieve L1 regularization through the use of a single weighting rule (regularization strength) across different sub-bands [1]. Since the level of sparsity varies across sub-bands, it has been shown that iteratively adapting the individual regularization strength for each sub-band can improve the recovery process [2]. However, levels of sparsity may vary significantly not only *between* but also *within* sub-bands, and taking advantage of this finer-grained variation may further improve reconstruction results, especially in scenarios where severe motion is present. In this work, we demonstrate that the use of a patch-based iteratively reweighted approach, in which regularization strength is adapted for each spatiotemporal patch in the transformed domain, can improve image reconstruction of exercise stress cardiac images relative to standard compressive recovery techniques.

## Methods

Exercise stress cine images in the long-axis orientation were acquired from three healthy volunteer on a 1.5T (Avanto, Siemens) scanner with a 32-channel body coil array at acceleration rate 6 using a VISTA [3] sampling pattern. Acquired data were reconstructed using a SENSE-based reconstruction with L1 regularization in the 3D spatiotemporal discrete wavelet domain. Two approaches were used for adjusting L1 regularization in the sparse domain. In the first approach (**FSW** - fixed single weighting), a fixed weighting rule was used across all sub-bands. In the second approach (**APBW** - adaptive patch-based weighting), sub-bands were divided into 2 × 2 × 2 patches and weighting rules were calculated for each patch using an adaptive method [2]. The total number of iterations was kept fixed to 150, and adaptive weights were recalculated for each iteration.

## Results

**Figure 1:**
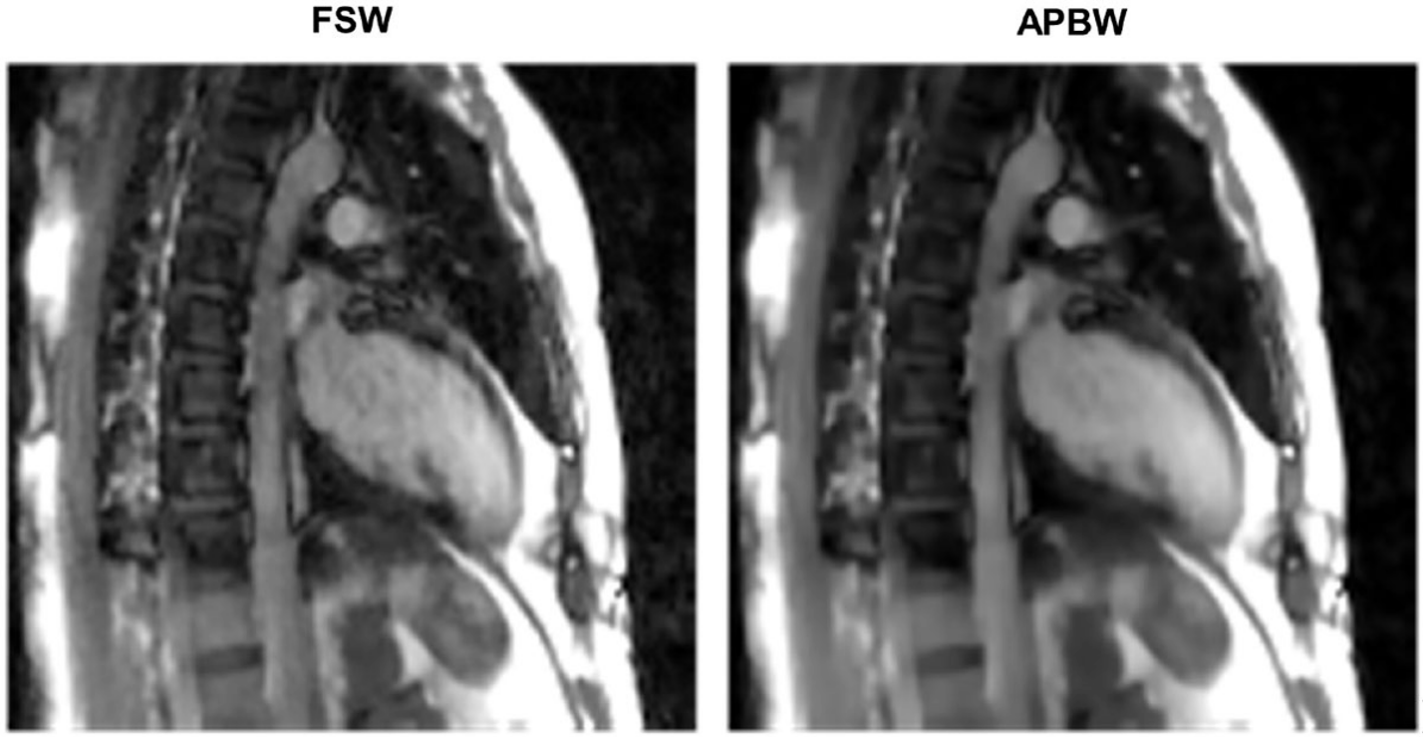
**Example images for each weighting method**. **APBW** shows reduced artifact level compared to **FSW**.

Figure 1 shows example images for each of the two methods. Figure 2 shows temporal profiles for each method along the line indicated. The use of adaptive patch-based weighting helps reduce artifact levels and improve sharpness of dynamic edges (Figure 2, arrow).

**Figure:**
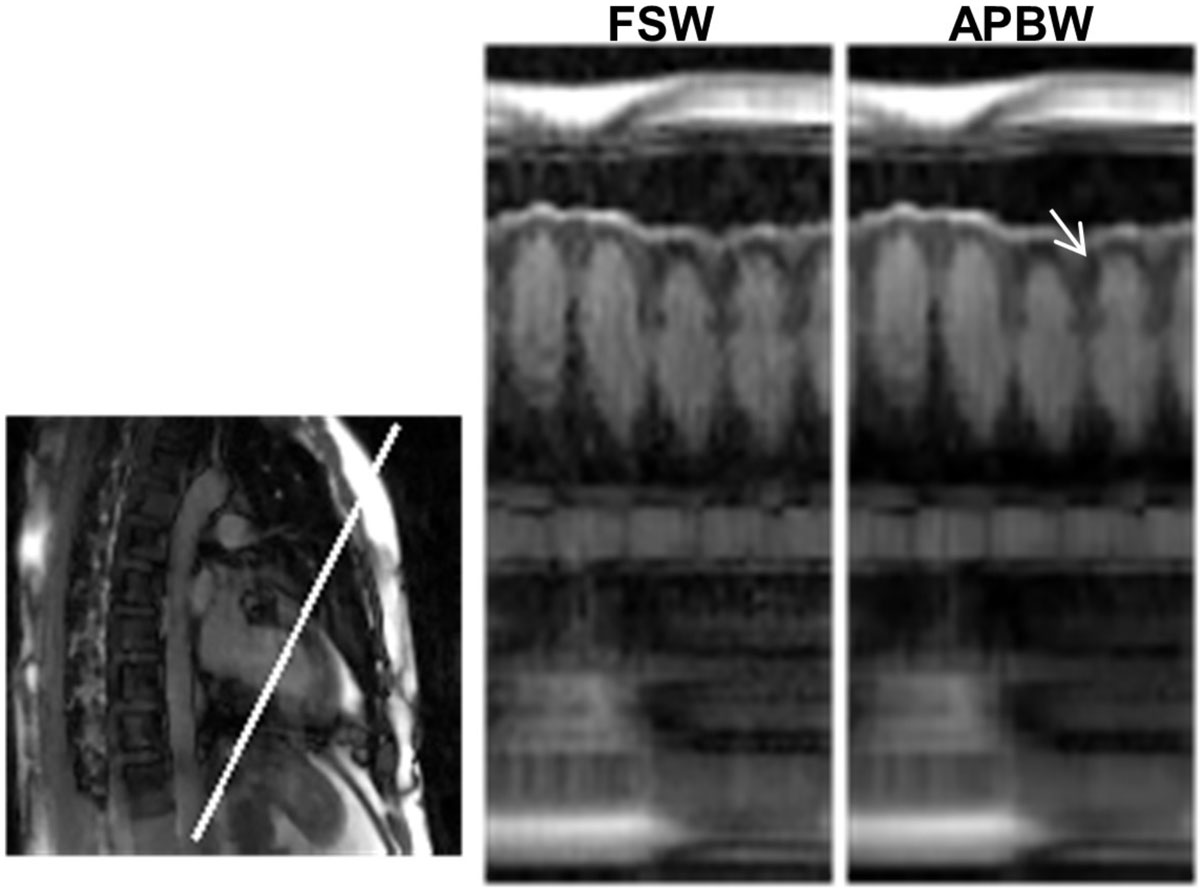
Figure 2

## Conclusions

Patch-based, iteratively-reweighted compressive recovery techniques can be used to take advantage of structured sparsity in exercise stress cardiac MRI, leading to improved image quality compared to standard techniques.
